# Rho GTPases in Model Systems

**DOI:** 10.3390/cells12050779

**Published:** 2023-03-01

**Authors:** Vedrana Filić, Igor Weber

**Affiliations:** Division of Molecular Biology, Ruđer Bošković Institute, Bijenička 54, HR-10000 Zagreb, Croatia

Since the discovery of their role in the regulation of actin cytoskeleton 30 years ago, Rho GTPases have taken center stage in cell motility research. Over time, it has become clear that these “molecular switches” are also involved in the regulation of other cellular processes, such as cell polarization, cell differentiation and growth, membrane trafficking, and transcriptional regulation. The biological importance of Rho GTPases is exemplified by the fact that mammalian cells invest more than 150 genes that encode their direct regulators. Rho GTPases are essential signaling molecules in mammals and across the whole eukaryotic domain. This Special Issue of *Cells* represents a collection of review articles and original research papers covering Rho GTPases in model organisms and cell culture systems. It can be roughly divided into sections devoted to evolutionary aspects, screening and mechanistic approaches, and the roles of Rho GTPases in mammalian physiology and pathology.

Evolutionary insight into the functional roles of Rho GTPases in simple model organisms can provide an invaluable perspective on their universal biological importance. Current knowledge and recent advances on how the fission yeasts Rho family GTPases regulate essential physiological processes, such as morphogenesis and polarity, cellular integrity, cytokinesis, and cellular differentiation, are presented by Vicente-Soler et al. [1]. Although the genetic tools available in yeast are still unrivaled, the physiology of the actin cytoskeleton in these organisms is quite specific and distinct from metazoan cells. A much closer match is provided by the highly motile cells of amoebozoan protists, which share the composition and dynamics of the transient actin-based structures, in particular with mammalian cells of the hematopoietic lineage. Filić et al. provide an overview of the Rho signaling pathways that regulate the actin dynamics in *Dictyostelium* and compare them with similar signaling networks in mammals [2]. Interestingly, although phylogenic algorithms do not identify strict homologies between Rho GTPases in the two groups, comprehensive functional studies established that canonical mammalian representatives, Rho, Rac and Cdc42, obviously have their functional analogues in *Dictyostelium*.

Moving on into the realm of multicellularity, a review by Beljan et al. represents a compilation of the current knowledge concerning Rho-family GTPases in non-bilaterian animals, the available experimental data regarding their biochemical characteristics and functions, and an original bioinformatics analysis of their relationship with metazoan counterparts [3]. Their findings provide a general insight into the evolutionary history of Rho-family GTPases in simple animals and support the notion that the ancestor of all animals probably contained Rho, Rac and Cdc42 homologs. Rho proteins of plants (ROPs) form a specific clade of Rho GTPases, which are involved in plant immunity and their susceptibility to diseases. In their review, Engelhard et al. summarize central concepts of Rho signaling in disease and immunity of plants and briefly compare them to important findings in the mammalian research field [4]. Interesting similarities emerge, as follows: while invasive fungal pathogens may co-opt the function of ROPs for manipulation of the cytoskeleton that promotes pathogenic colonization, mammalian bacterial pathogens also initiate effector-triggered susceptibility for cell invasion via Rho GTPases. Zebrafish, as model vertebrates, offer a unique opportunity to explore the spatial and temporal dynamics of Rho GTPases within a complex environment at a level of detail unachievable in any other organism. Boueid et al. present a compilation of examples where the roles of Rho, Rac and Cdc42 in cell motility, developmental processes, angiogenesis, neural system and pathological processes in zebrafish were investigated using a set of powerful tools to follow and locally modulate Rho GTPases signaling and their function in real time, combined with rapidly evolving imaging and genetic techniques [5].

Given the large complexity involved in the signaling networks centered on Rho GTPases, system-level research is required to fully grasp the extent of their biological roles and regulation. In their review article, Dahmene et al. highlight the recent large-scale studies, including proteomic approaches to map the full repertoire of Rho GTPases and Rho regulators protein interactions and high throughput screening strategies that unraveled new roles for understudied family members by using cell culture models and mouse embryos [6]. An example of the high throughput screening approach is provided by Long et al., who developed and applied an image-based high-content screen using RNA interference to systematically perturb each of the 21 Rho family members and assess their importance to the overall organization of the Golgi complex [7]. Their analysis revealed previously unreported roles for two atypical Rho family members, RhoBTB1 and RhoBTB3, in the endomembrane traffic events. In addition to their localization at the plasma membrane and endomembranes, recent research demonstrates that active pools of different Rho GTPases also localize to the nucleus. Navarro-Lérida et al. discuss how the modulation of Rho GTPases driven by post-translation modifications provides a versatile mechanism for their compartmentalization and functional regulation [8]. They stress that understanding how the subcellular sorting of active small GTPase pools occurs and what its functional significance is will contribute to the exploration of Rho GTPases as important therapeutic targets in cancer and other disorders.

Indeed, it is becoming increasingly clear that small Rho GTPases play essential roles in human physiology and pathology. In their review article, Sarowar and Grabrucker discuss the role of three archetypical Rho GTPases in the modulation of dendritic spine morphogenesis in the amygdala, the core brain region associated with fear learning and conditioning [9]. Dendritic spines are tiny, dynamic, and heterogeneous actin-rich protrusions on the surface of neuronal dendrites that receive input from an axon at the synapse. RhoA inhibits dendritic growth and dynamics, while Cdc42 and Rac1 promote them. By doing so, Rho GTPases and some of their modulators expressed in the amygdala play an important role in fear-related processes. Another example of the influence of Rho signaling on synaptic plasticity is provided by Figiel and colleagues [10]. Synaptic remodeling mediated by matrix metalloproteinase 9 (MMP-9) is essential for long-term memory formation. MMP-9 may contribute to the dynamic remodeling of structural and functional plasticity by cleaving ECM components and cell adhesion molecules. Rho GTPases seem to be downstream effectors of MMP-9, and the authors review current knowledge on their roles in MMP-9-dependent signaling pathways in the brain, with emphasis on the influence of their post-translational modifications.

Mao et al. explored the function of Rho GTPases during phagocytosis of spent photoreceptor outer segment fragments by retinal pigment epithelial (RPE) cells, which is essential for visual function [11]. This process is mediated by Mer tyrosine kinase (MerTK) receptor signaling, and MerTK mutations cause complete blindness in early adulthood, for which widely applicable therapy is still unavailable. In their research, the authors show that efficient RPE phagocytosis requires the activation of Rac1 and the simultaneous suppression of RhoA activity downstream from MerTK. In MerTK-deficient RPE cells, elevated RhoA activity blocks phagocytic cup formation. However, inhibition of RhoA downstream effector ROCK is sufficient to restore the phagocytic capacity of MerTK-deficient RPE. Since ROCK inhibitors are already approved for common, long-term use for ocular disease, this study supports future efforts toward their use in simple therapy, possibly as eye drops. Veluthakal and Thurmond systematically summarize the role of different Ras superfamily GTPases in the normal functioning of pancreatic islet β-cells [12]. Pancreatic islet β-cells take up glucose and initiate glucose metabolism that, via a plethora of signaling events, induces insulin granule exocytosis. The authors review the roles of Rho, Arf and Rab family members in the islet insulin secretory process and describe how the altered activity of GTPases can lead to β-cell dysfunction.

Considering the important roles that Rho GTPases play in the regulation of physiological processes, it is no surprise that their altered activities significantly contribute to numerous pathological conditions. Humphries et al. provide an overview of the current understanding of the regulation and functions of Rho GTPases specifically in breast cancer [13]. They show that, similarly to findings in other tumors, there are conflicting data concerning the role of Rho GTPases in breast cancer. However, the prevalent notion is that increased activation of Rho GTPases has tumor-promoting roles in breast cancer initiation, metastatic progression, chemoresistance, and radioresistance. Furthermore, Rho GTPases have important roles in diseases whose major contributor is chronic inflammation. Kilian et al. summarize the role of RhoA in injury- and stress-induced signaling from damaged cardiomyocytes to immune cells, which leads to immune cell activation, chronic inflammation and cardiac disease progression [14]. They discuss the possible roles of RhoA signaling in cardiomyocytes, macrophages, neutrophils, and dendritic cells, which are important in the pathogenesis and progression of cardiac dysfunction. Atherosclerosis is another example of a chronic inflammatory immune-mediated condition implicated in the pathogenesis of coronary artery disease, a major cause of mortality worldwide. Lee et al. highlight the role of Rac-mediated inflammatory signaling in the mechanisms driving atherosclerotic calcification [15]. They also discuss the off-target effects of statins, the most widely used therapy for hypercholesterolemia, on Rac immune signaling. Inflammation also has a crucial role in the development of gastrointestinal diseases, such as inflammatory bowel disease (IBD) and colorectal cancer. IBD is marked by the uncontrolled activation of immune cells and alterations to intestinal epithelial cells (IECs), characterized by increased tight junction permeability and altered cytoskeletal rearrangements. Pradhan et al. summarize the current knowledge on the roles of classical Rho GTPases in the context of intestinal homeostasis and disease, focusing on IECs and T cells [16]. Finally, Hahmeyer and da Silva-Santos provide a comprehensive overview of the current knowledge about the role of Rho signaling pathways in sepsis, a life-threatening organ dysfunction caused by a dysregulated response to infection [17]. They describe how Rho proteins, mainly Rac1 and RhoA, are involved in the development of sepsis-specific symptoms in different systems and cells, including the endothelium, vessels, and the heart.
